# Supplementary material for: SARS-CoV-2 (COVID-19 pandemic) in Nigeria: Multi-institutional survey of knowledge, practices and perception amongst undergraduate veterinary medical students
Source: PLoS One. 2021 Mar 15;16(3):e0248189. doi: 10.1371/journal.pone.0248189 (PMC7959340; doi:10.1371/journal.pone.0248189)
Supplement: S1 File — (DOCX) [file pone.0248189.s001.docx]

**Title: COVID 19 pandemic in Nigeria: Knowledge, Practices, and Perception of undergraduate veterinary medical students**

This questionnaire is aimed at gathering information on your knowledge, practices and perception towards the COVID 19 pandemic. Also, we will be happy to know the state of your mental wellbeing presently.

We will be grateful if you may kindly provide the required information to the best of your ability/knowledge. Information collected will be kept confidential and for advancement of knowledge and intervention purposes only.

The filling of the questionnaire will take approximately 8 minutes of your time

Many thanks for your kind support

**Section A: Demographics**

1. Age as at last birthday (in years):
2. Sex: **Female Male**
3. Religion: **Christianity Islam Traditional Others**
4. Name of University:

**University of Ibadan,**

**Ahmadu Bello University,**

**Usman Fodyio University,**

**University of Maiduguri,**

**University of Nigeria,**

**FUA, Markurdi,**

**FUNAAB,**

**University of Abuja,**

**University of Ilorin,**

**Micheal Okpara University of Agriculture**

**UniJos**

1. School Year:

**DVM 1**

**DVM 2**

**DVM 3**

**DVM 4**

**DVM 5**

1. State of residence during lockdown:
2. *Number household members ----------------------------
3. Type of lockdown instituted: Partial Total Not sure

**Section B: Knowledge on COVID 19**

1. Source of information on COVID 19 (Tick all that applies)

Television /radio

Newspapers

Internet (including social media e.g., Facebook)

Friends

Parents

School

Others (Specify):

1. COVID 19 is caused by

Virus

Protozoan

Bacteria

Fungi

Others

1. Incubation Period of the disease is

1-2 days

3-7 days

2-14 days

15-28 days

1-2 months

1. COVID- 19 is similar to:

Common flu

MERS-CoV

SARS-CoV

Don’t know

5. COVID 19 was first reported in

USA

China

Singapore

Italy

UK

Not sure

6. Identify as many as possible the common symptoms of COVID 19

Fever

Dry cough

Runny nose

Shortness of breath

Joint/Muscle ache

Red eyes

Loss of taste

Loss of smell

Diarrhea/vomiting

Wet cough

Fatigue

7. COVID 19 can be transmitted through

Direct contact with infected person

Air droplet

Indirect contact such as contaminated surfaces

Sexual contacts

Fecal-Oral

Handshake

Kissing

8. It is possible to have COVID 19 and not show symptoms

Yes

No

I don’t know

9. What can kill the virus (select all that applies)

Alcohol-based sanitizers

Soap/detergents

Wipes

Water alone

Sunlight

High temperature

I don’t know

10. Pets have been scientifically proven to transmit COVID 19

Yes

No

I don’t know

11. There is a cure for COVID 19

Yes

No

I don’t know

**Section C: Practices towards COVID 19**

**Never, sometimes, all the time**

1. I observed the stay-at-home order
2. I wash my hands with soap and water or use sanitizer
3. I avoid crowded places
4. I avoid handshake and touching people
5. I do not touch my face or nose
6. I wear a facemask whenever I leave the house
7. I visit friends and/or attend parties
8. I maintain good personal hygiene
9. I engage in self-medication to prevent contracting COVID 19
10. I maintain physical/social distance from other people.

**Section D: Perception: Strongly Agree, Agree, Undecided, Disagree, Strongly Disagree**

1. I have never seen anyone with COVID 19, so the disease is a scam.
2. It is the disease of the elite. No record of serious incidence among the masses
3. I cannot remember what I have been taught in school and worried my academic performance will be affected negatively when school resumes
4. If schools resume, there is a high risk of contracting the disease
5. The pandemic has affected my interest in the veterinarian profession
6. Nigerian universities are well equipped with various online tools, platforms and facilities for virtual learning
7. I have internet facilities to access online courses related to my field
8. Time spent on social media is more than studying/enrolling for online programs in my field
9. This pandemic provides the opportunity to start a relationship or get married
10. People with COVID 19 are highly stigmatized
11. I am optimistic the pandemic will soon be over
12. The COVID-19 pandemic has made me feel depressed]
