# Supplementary material for: SARS-CoV-2 (COVID-19 pandemic) in Nigeria: Multi-institutional survey of knowledge, practices and perception amongst undergraduate veterinary medical students
Source: PLoS One. 2021 Mar 15;16(3):e0248189. doi: 10.1371/journal.pone.0248189 (PMC7959340; doi:10.1371/journal.pone.0248189)
Supplement: Attachment — Submitted filename: Plos one review.docx [file pone.0248189.s002.docx]

Thank you for giving me the opportunity to review this manuscript in which the authors reported a Multi institutional survey of knowledge, practices and perception of SARS-CoV-1 2 (COVID-19 pandemic) amongst undergraduate veterinary medical students in Nigeria.

The method employed was appropriate and the study was well conducted and ethically sound. The study is also relevant as it gives insight on the knowledge & Practices of this important Pandemic in the study population

However, the sampling technique was not well explained and

This paper is publishable after making a few corrections and clarification as pointed in comment to authors below

Many thanks

AD Geidam

Consultant Obstetrician and Gynaecologist.

This study is well conducted, ethically sound and relevant as it provides insight on the knowledge & Practices of this important Pandemic in the study population. I have a few comments and suggestions.

1) Line 78 “However, the numbers of cases and deaths are still on the rise…….” is this statement correct as at the time of writing????
2) Line 127-128 “At least, it was ensured one veterinary school was representative of each of the geopolitical zones in Nigeria” …… this statement is redundant as the only university that was not represented was the one that was excluded.
3) Lines 129-131 “A total of 385 participants were needed considering ………. with an acceptable error of 5%” ……….. were needed to achieve what??? Power????
4) Lines 131-132 “ 10% of the sample size was added to give a total of 423 participants …. 10% attrition = 478
5) Although it was stated in line 132 that ……” For student recruitment, a purposive and chain referral sampling techniques was performed” give details of how the sampling was done. What is the end point? How was the sample size distributed for the variuod schools etc
6) Line 150 “responses were scored” How???
7) Lines 190-192 “The levels of measured outcomes were expressed as mean and standard deviation (Mean ± SD)”……. Even if they are not normally distributed????
8) Line 197 “Variables significant at p ≤ 0.25 were processed further by the logistic regression” what inform the use of this cut off
9) I don’t know if the 95% CI in table 1 is necessary
10) The OR in table 5is it crude or adjusted????
