# Supplementary material for: SARS-CoV-2 (COVID-19 pandemic) in Nigeria: Multi-institutional survey of knowledge, practices and perception amongst undergraduate veterinary medical students
Source: PLoS One. 2021 Mar 15;16(3):e0248189. doi: 10.1371/journal.pone.0248189 (PMC7959340; doi:10.1371/journal.pone.0248189)
Supplement: Attachment — Submitted filename: Responses to reviewers comments (PLoS ONE).docx [file pone.0248189.s003.docx]

**RESPONSES TO EDITOR’S AND REVIEWERS’ COMMENTS**

We are grateful for the opportunity given to revise our manuscript. The suggestions made by the reviewers were found very useful and we thank them.

Below are our responses to the comments, accordingly.

| **S/N** | **Location/lines revised** | **Reviewer’s comment** | **Remarks(Authors’ responses)** |
| --- | --- | --- | --- |
| **Plos One_ Editor’s queries/comments**   1. **there are specific methodological issues to be addressed before acceptance. For example, the power calculation, the recruitment procedures, decisions on cutpoints and scale transformation, and the rational for the multivariate analysis are not clear. Also, the discussion of the limitations of the approach utilized for the study has also not been broadly contextualized**   **Authors’ responses**  **Power calculation:** The sample size for this survey was calculated based on the assumptions that poor level of knowledge and practices among respondents was 50%, an absolute precision of 95% confidence interval, and an acceptable error of 5%. Using Working in Epidemiology (WinEpi v.2.0), a total of 385 participants was estimated while a 10% non-contingency was added to make up for non-response giving a minimum target sample size of 423 participants (Lines 133 – 140).  **Recruitment procedures:** The total sample size was divided equally among the veterinary schools (43 students per university). We purposively recruited a minimum of nine at each level (DVM 1-DVM 5) across the participating universities and participation was made voluntary. We employed an online survey due to the COVID pandemic and lockdown policy in the country as at the time of study. Purposive and convenience chain referral sampling technique was performed for student recruitment. Briefly, prior commencement of the study, the national president of the Association of Veterinary Medical Students (AVMS) and his counterparts at the various university chapters were contacted. A detailed information on the project focus, aims, and plans for student recruitment were discussed over several online meetings. Following their consent, invitations were sent to students nationwide to participate in the study using the WhatsApp platforms of the Association of Veterinary Medical Students (AVMS) of the various university chapters. The link to the online survey questionnaire was included in the sent invitations and a brief description of the purpose of the study was provided. Also, class coordinators at various levels (DVM 1 to 5) were further assigned to share the questionnaire on their respective class WhatsApp platforms, while three of the authors were delegated to follow up on this process to enhance participation. (lines 140 -157)  **Determination of cut-off points:** The scores were thereafter converted to percentages, and based on the students’ mean scores in knowledge and practices categories, cut-off points for good / satisfactory were set at ≥70%, while those below (<70%) were considered to have poor/ unsatisfactory levels. These cut-off points were so set since it is expected that such students on medical profession should have basic knowledge and demonstrate practices towards issues related to health. Besides, such cut-off points had earlier been employed in a similar study (lines 229 – 234)  **The rational for the multivariate analysis are not clear:** A stepwise forward likelihood multivariate analysis was conducted in this study to identify predictive variables that influenced the knowledge and practices among Nigerian students towards COVID -19 prevention. This was chosen in order to avoid variation in results from individual univariate tests of different measures due to random chance. (lines 239 – 244)  **Limitations of the approach utilized for the study:**  Some limitations of the approach utilized in the study were identified. Firstly, the introduction of an enrolment and reporting biases may have resulted as this is an online survey and data collected were self – reported. We observed shortcoming in recruitment approach may have reflected in the uneven distribution of student respondents from the different universities. Secondly, the poor internet accessibility or connectivity in the country (which may have varied from one institution location to another) and lack of funds to purchase data as complained by some students may have contributed to the lack of access to online questionnaire and participation. Thirdly, a convenience, non- probabilistic sampling technique was used, and generalization of our findings to the entire veterinary student populations in the country may be difficult and should be interpreted with caution. However, we believe the overall response rate which was above the minimum estimated sample size is sufficient to eliminate or reduce these biases. (Lines 465 – 480)   2. **Please ensure that your manuscript meets PLOS ONE's style requirements, including those for file naming. The PLOS ONE style templates can be found at** **https://journals.plos.org/plosone/s/file?id=wjVg/PLOSOne_formatting_sample_main_body.pdf and**   **https://journals.plos.org/plosone/s/file?id=ba62/PLOSOne_formatting_sample_title_authors_affiliations.pdf**  **Authors’ Response**  Manuscript has been formatted according to the formatting samples provided   3. **Thank you for including your ethics statement:** **Please provide additional details regarding participant consent. In the ethics statement in the Methods and online submission information, please ensure that you have specified what type you obtained (for instance, written or verbal, and if verbal, how it was documented and witnessed).**   **Authors’ response**  Participants’ consent was obtained verbally and witnessed by the class coordinators of different levels (HOC- DVM 1 to 5). (Lines 210-211)   4. **Data Availability**   **Authors’ response**  There is no ethical or legal restrictions to accessing our data. The datasets have been deposited in Mendeley data. Adebowale, Oluwawemimo; Adenubi, Olubukola; Adesokan, Hezekiah; Oloye, Abimbola; Bankole, Noah; Fadipe, Oladotun; Ayo-Ajayi, Oluwatoyin ; Akinloye, Adebayo (2020), “SARS-CoV-2 (COVID-19 Pandemic) in Nigeria: Multi-institutional Survey of Knowledge, Practices and Perception Amongst Undergraduate Veterinary Medical Students”, Mendeley Data, V1, doi: 10.17632/jy7hh77f8c.1  http://dx.doi.org/10.17632/jy7hh77f8c.1   5. **We note that [Figure(s) 1] in your submission contain [map/satellite] images which may be copyrighted. All PLOS content is published under the Creative Commons Attribution License (CC BY 4.0), which means that the manuscript, images, and Supporting Information files will be freely available online, and any third party is permitted to access, download, copy, distribute, and use these materials in any way, even commercially, with proper attribution. For these reasons, we cannot publish previously copyrighted maps or satellite images created using proprietary data, such as Google software (Google Maps, Street View, and Earth). For more information, see our copyright guidelines:** [**http://journals.plos.org/plosone/s/licenses-and-copyright**](http://journals.plos.org/plosone/s/licenses-and-copyright)**.**   **Authors’ response**  A new figure has been provided by a mapping expert. The shapefile used to generate the figure was downloaded from [www.diva.gis.org](http://www.diva.gis.org), initiated, analysed and produced with ArcMap 10.5 software. Please, we are happy to have the figure removed if this still does not meet your copyright policy | | | |
| **Response to Reviewer 1** | | | |
| **Introduction** | | | |
| 1 | Line 78 | **“However, the numbers of cases and deaths are still on the rise…….” is this statement correct as at the time of writing????** | Rephrased. Figures to show rise in cases from commencement to the end of the study are revised within the manuscript. Now reads  “According to the NCDC, more than 286,000 tests, 43,537 confirmed positive cases, 22,567 active cases, 20,087 discharges and 883 human deaths were reported as at the commencement of this study, August 1^st^, 2020 [3] across 36 states in the country, including the Federal Capital Territory (FCT), Abuja. However, the numbers of cases and deaths are on the rise with 56,177 confirmed cases, 1,078 fatalities as at the time of article submission (September 12^th^, 2020). Lines 83 -87. |
| **Materials and Methods** | | | |
| 2 | Line 127-128 | “At least, it was ensured one veterinary school was representative of each of the geopolitical zones in Nigeria” …… this statement is redundant as the only university that was not represented was the one that was excluded | This is right Sir. This sentence has been deleted. Many thanks |
| 3 | Lines 129-131 | “A total of 385 participants were needed considering ………. with an acceptable error of 5%” ……….. were needed to achieve what??? Power???? | This has been addressed at the Editor’s comment section. The lines now reads  “The sample size for this survey was calculated based on the assumptions that poor level of knowledge and practices among respondents was 50%, an absolute precision of 95% confidence interval, and an acceptable error of 5%. Using Working in Epidemiology (WinEpi v.2.0), a total of 385 participants was estimated for the survey. a total of 385 participants was estimated while a 10% non-contingency was added to make up for non-response giving a minimum target sample size of 423 participants (lines 133 – 140) |
| 4 | Lines 131-132 | “ 10% of the sample size was added to give a total of 423 participants …. 10% attrition = 478 | The authors have done a recalculation and confirmed a 10% attribution = 423.5 |
| 5 | Line 132 | Although it was stated in line 132 that ……” For student recruitment, a purposive and chain referral sampling techniques was performed” give details of how the sampling was done. What is the end point? How was the sample size distributed for the variuod schools etc | **Recruitment procedures:** Due to the COVID pandemic and lockdown policy in the country as at the time of study, an online survey was employed, and purposive and convenience chain referral sampling technique was performed for student recruitment. Briefly, prior commencement of the study, the national president of the Association of Veterinary Medical Students (AVMS) and his counterparts at the various university chapters were contacted. A detailed information on the project focus, aims, benefits and plans for student recruitment were discussed over several online meetings. Following their consent, invitationswere sent to students nationwide to participate in the study using the WhatsApp platforms of the Association of Veterinary Medical Students (AVMS) of the various university chapters. The link to the online survey questionnaire was included in the sent invitations and a brief description of the purpose of the study was provided. Also, class coordinators at various levels (DVM 1 to 5) were further assigned to share the questionnaire on their respective class WhatsApp platforms , while three of the authors were delegated to follow up on this process to enhance participation.. (Lines 143 – 157)  **Sampling End point:** Where undergraduate veterinary students from various levels of DVM (1 to 5)  **Sample size distribution:** The total sample size was divided equally among the veterinary schools (43 students per university). We purposively recruited a minimum of 9 at each level (DVM 1-DVM 5) across the participating universities and participation was made voluntary. (Lines 140 -143) |
| 6 | Line 150 | “responses were scored” How??? | The responses were scored as “1” for the “correct answer” and “0” for an “incorrect” or “I don’t know” response.  ( Lines 177 – 179) |
| 7 | Lines 190-192 | “The levels of measured outcomes were expressed as mean and standard deviation (Mean ± SD)”……. Even if they are not normally distributed???? | The measured outcomes were tested for normality using the using Kolmogorov-Smirnov (> 0.05), which informed our use of (Mean ± SD) (lines 229 -230) |
| 8 | Line 197 | “Variables significant at p ≤ 0.25 were processed further by the logistic regression” what inform the use of this cut off | The decision for liberal p-value (p≤ 0.25) at this step was to ensure important potential predictor/risk variables were included in the model (Bursac et al. 2008). |
| **Results** | | | |
| 9 | Table 1 | I don’t know if the 95% CI in table 1 is necessary | This column has been deleted |
| 10 | Table 5 | The OR in table 5is it crude or adjusted???? | Adjusted Odds Ratio and changes have been made on the table with footnotes |
| **Conclusions** | | | |
| 11 | Lines 147 -148 | The first sentence in the conclusion is not derived from your data | Thank you for this observation. This sentence has been deleted. |
| **Reviewer 2** | | | |
| **Materials and Methods** | | | |
| 12 | Line 130 | In the methods, the required sample size was about 400. This is a standard for a KAP Questionnaire study. However, the sample size does not require response rate estimates. It requires the expected differences to be detected. Please reconsider this note in the writing up. | This has been noted for action. And has been addressed in the Editor and first reviewers sections. Changes have also been made in the manuscript ( Lines 133 -140) |
| 13 | Line 133 | “For student recruitment, a purposive and chain referral sampling techniques was performed”. A brief description may be necessary as this is not a standard sampling method used for questionnaires. | This is a non – probabilistic method of sampling, which had to be used due to the COVID 19 lockdown policy at the time of study  Meanwhile, a detailed process for student recruitment has been provided in the Editor and First reviewer comment sections. Ammendments have also been made in the write up.  **Now reads:** “Due to the COVID pandemic and lockdown policy in the country as at the time of study, an online survey was employed, and purposive and convenience chain referral sampling technique was performed for student recruitment. Briefly, prior commencement of the study, the national president of the Association of Veterinary Medical Students (AVMS) and his counterparts at the various university chapters were contacted. A detailed information on the project focus, aims, benefits and plans for student recruitment were discussed over several online meetings. Following their consent, invitationswere sent to students nationwide to participate in the study using the WhatsApp platforms of the Association of Veterinary Medical Students (AVMS) of the various university chapters. The link to the online survey questionnaire was included in the sent invitations and a brief description of the purpose of the study was provided. Also, class coordinators at various levels (DVM 1 to 5) were further assigned to share the questionnaire on their respective class WhatsApp platforms , while three of the authors were delegated to follow up on this process to enhance participation”. ((Lines 143 – 157) |
| 14 | Line 145 | these questions are not clearly presented. Were they measured on a likert-scale? Yes/no? each Question had the same responses as for all the questions in the survey? Where did the 31 total sum come from? Summing all responses? Were they in the same direction? | The different sections i.e. knowledge, practices and perceptions had different scales of measurements. The practices and perception were measured on a Likert scale, while Knowledge was on a dichotomous response Yes/No.  Each question does not have same response and this has been elaborated more in the materials and methods section as suggested.  The summing up of 31 were scores summed for all the responses in the section  **Ammendment now reads:**  “To measure students’ general knowledge about COVID-19, an 11-item questionnaire that  assessed the source of information about COVID-19 and general awareness questions were provided in section B. Question one addressed the various sources of updates and information on the pandemic were requested. The other ten questions focused on clinical presentation, transmission, prevention, and control strategies of COVID-19. Question asked included the cause of COVID-19 infection, incubation period, risk conditions, country of the first outbreak, modes of transmission, identification of common symptoms of the infection, if it was possible to have asymptomatic individuals, and methods of prevention, control and treatment. Each correct answer weights 1 point and 0 for incorrect or I don’t know answers. Score for each responses was summed up to give 31 points.  ”. (Lines 170 – 179) |
| 15 | Line 170 | This was an online survey. Anyone could have answered if respondents received the link. How can you be sure it was only filled by Vet Med students? | With due diligence it was ensured that only veterinary students responded to the survey. We first had series of meetings with all presidents of the association of the veterinary medical students who ensured links were shared with the class coordinators at each veterinary level in their respective universities (DVM 2 to 5). The class coordinators were then assigned to strictly share the link/questionnaire on respective veterinary class whatapp platforms and closely monitored by a few authors to ensure participation and that only veterinary students filled the questionnaire. |
| 16 | Line 192 | Why was this cutoff point selected? Why not, for example, 75%? Or 80%? | The mean scores for both knowledge and practice levels were 73.4% and 80.3%. The cut off of 70% was used to establish good and satisfactory knowledge and practice levels according to Kaliyaperumal KI. Guideline for conducting a knowledge, attitude and practice (KAP) study. AECS Illumin 2004; 4(1): 7–9. The reference has been included in the manuscript |
| 17 | Lines 189 - 190 | Why were the responses converted from a likert scale into 0 and 1? This is a descriptive study and readers may want to see the actual scores? Such conversion will limit the data and affect the comparison between groups of subjects | We apologize for this mix up and has been rectified in lines 216 – 220.  It was only the knowledge responses that were coded as “0” or “1” for “wrong” or “correct” responses  **The adjustment now reads**  “To evaluate the knowledge level of respondents, a numeric pattern of scoring was used by giving a score of “1” for the “correct answer” and “0” for an “incorrect” or “I don’t know” response. Similarly, the practice level was assessed by giving scores of “1” for the “never” and “2” for “sometimes” and 3 for “all the time” responses ” (lines 224 -228) |
| **Results** | | | |
| 18 | Table 1 | Some variables in table 1 could be collapsed to make more sense of the presented data. | We have deleted the 95% CI column as suggested by reviewer **1. However, the authors suggest it is would make more sense to give a detailed description of all the variables collected by the questionnaire in the demographic section questionnaire.** |
| 19 | Figure | Figures are not clear and not of any added value. | We have addressed the problem with figure 1 that is the mapping of participating schools. We have informed the editor in our response that we are happy yo get this removed if it was yet to addresses the copyright concern.  Figures 2& 3 have been removed because these do not provide any added value as you have rightly suggested.  The authors however are of the opinion the figures 4 (now 2) and 5(now 3) should remain as they provide detailed description and important information to the responses to practices and perceptions of students in relation to the pandemic. The resolution has been adjusted to the best of out ability and new figures will be uploaded |
| 20 | Line 219 | some of the percentages presented in this section are scary. Especially for Vet Med students who should be more informed about the disease. Knowledge questions should be properly presented in the methods.  Some of the knowledge points in table 2 are still controversial. How this was decided to be true or false is still under scientific discussion. | The knowledge questions have been provided in details in the materials and methods as suggested. Please refer to item 14.  We should bear in mind that this study involved students in different phases of their DVM programme. Some studenets especially those in the pre clinical years who have not been exposed to trainings in epidemiology of infectious disesaes and control could properly have a lowere knowledge capacity compared to clinical students. This was confirmed and explained further in the logistic regression analysis that predicted good knowledge levels among clinical studnts than non clinical ones. This actually to us is not suprising because of their different levels of clinical exposures i.e. infectious disease controland epidemiology courses  Our judgements were based on all scientific evidences or studies available on the epidemiology of COVID 19. For instance as a veterinarian, there is still till date no scientific evidence to show pets can transmit the virus. So, we did not expect a positive response to this question as at the time of the study. |
| 21 | Table 3: | a p value above 0.05 is not statistically significant but could be used to decide on variables for regression models. Please modify the table notes accordingly. | Many thanks Sir. This has been modified |
| 22 |  | Also, university name and region may be collinear with each others. Having them in the same model will introduce noise and redundancy. | Great thanks for this observation. We have deleted the university name as a variable. |
| 23 |  | Also, sample size calculations are conducted for a KAP study where a prevalence is presented.  Conducting a logistic regression may not be a good idea especially with the number within each cell of the table. Collapsing the categories within each variable is needed to be able to conduct a logistic regression analysis. | This has been addressed at the Editor’s comment section (Item 3).  “The sample size for this survey was calculated based on the assumptions that poor level of knowledge and practices among respondents was 50%, an absolute precision of 95% confidence interval, and an acceptable error of 5%. Using Working in Epidemiology (WinEpi v.2.0), a total of 385 participants was estimated for the survey but to make up for non-response, a 10% non-contingency was added to to make up for non-response, giving a minimum target sample size of 423 participants  Many thanks for the suggestion. We have collapsed some categories within age groups as well as regions together and the data were re-analysed. |
