# Supplementary material for: SARS-CoV-2 (COVID-19 pandemic) in Nigeria: Multi-institutional survey of knowledge, practices and perception amongst undergraduate veterinary medical students
Source: PLoS One. 2021 Mar 15;16(3):e0248189. doi: 10.1371/journal.pone.0248189 (PMC7959340; doi:10.1371/journal.pone.0248189)
Supplement: Attachment — Submitted filename: RESPONSES TO EDITOR_ revision 2.docx [file pone.0248189.s004.docx]

**RESPONSES TO EDITOR’S AND REVIEWERS’ COMMENTS (REVISION 2)**

The authors are grateful for the comments and suggestions provided by the reviewers, which we found helpful and improved the quality of manuscript. Thank you Sirs

Below are our responses to the comments, accordingly.

| **S/N** | **Location/lines revised** | **Reviewer’s comment** | **Remarks(Authors’ responses)** |
| --- | --- | --- | --- |
| **Plos One_ Editor’s queries/comments** 1. **There are still concerns on methodological clarity of the manuscript and the external validity of the results. At present the methods is not sufficiently described to support independent replication of the study. Please clarify and include a statement in the manuscript to confirm that the online survey was based on the Checklist for Reporting Results of Internet E-Surveys (CHERRIES), and guidelines for good practice in the conduct and reporting of online research.** **Response:** Thank you and we have clarified and confirmed in the manuscript that the online survey was based on the checklist for reporting results of internet E – surveys (CHERRIES), and guidelines for good practice in the conduct and reporting of online research (201 -203). We also ensured it was stated in the manuscript the participation rate of more than 100.0% (lines 254 -255), that a minimum of 9 students were then conveniently sampled from each level (141-143), the completion of the online survey took about 8 minutes and designed to ensure duplicate entries was avoided by preventing users with the same IP address access to the survey twice access to the survey twice (197 -199), ethical approval and informed consent described, development and pre-testing of the questionnaire was mentioned, survey administration was also provided in detail including the type of e-survey, survey context, that it was voluntary for participant, incentives offered has been included (lines 157 – 158). 2. **It will be good if the authors, based on their local knowledge, provide an estimate of the total number of all the veterinary medical students (DV1 to DV5) in the study population (the 10 enlisted universities) that were eligible to participate in the study.** **This way the readers can make their independent judgment on the external validity of the results beyond the calculated sample size and the response rate.** **Response:** The enlisted number of universities in the study were 11. The total number of all the veterinary medical students eligible to participate in the study was 3724 (lines 131 -132). 3. The last sentence of the limitation section seems tenuous. It is ambiguous to claim that self-selection and respondent biases or issue of generalizability can be eliminated by having a response rate above the minimum estimated sample size. Using only limited number of participants in each school constitute a threat to the external validity of the results that is worth further discussion in the limitation section of the manuscript. **Response:** Thank you Sir. The authors quite agree that the last statement seems ambiguous and that generalizability cannot be eliminated by having a response rate above the minimum estimated sample size, coupled with other several limitations associated with the online survey e.g. the non-probabilistic sampling technique used. 1. We have further expatiated the limitation of getting low participation from some schools, which was attributed to inaccessibility to the internet or inability to afford money to purchase data and insufficient power supplies (common complaints by the students) 2. We recognise for the online survey, random sampling was also impossible so we resulted to convenience sampling and voluntary participation, which also may have introduced selection bias. Due to challenges peculiar to the country as well as this study, we are therefore cautious with generalizing the result or outcomes of study to both veterinary student populations and across contexts. **“**Some limitations of the approach utilized in the study were identified. The introduction of enrolment and reporting biases may have resulted from the online survey making it non-representative. For the study, randomization was impossible due to the national lockdown, which could have possibly eliminated some of the biases. The non- probabilistic sampling approach, which is convenience and voluntary may have contributed to in the uneven distribution (coverage and non-participation) of student respondents from the different universities investigated. Furthermore, the poor internet accessibility or connectivity in the country (which may have varied from one institution location to another) and lack of funds to purchase data as complained by some students may have contributed to the lack of access to online questionnaire and participation. We are therefore cautious in generalizing the sample findings to the whole veterinary student populations in the country due to these limitations”. 4. **If possible, the authors should also consider to include the flow chart of the participants’ recruitment process to clearly indicate how many participants from each school click on the survey link (how many did not attempt any question), how many started the survey (how many started but did not complete) and how many finally completed the survey.** **Response:** The data we have i.e. The total number of students that filled and completed the questionnaire, which is = 437. I have tried to include a flow chart as requested. The authors however, think since all participants accessed the link and completed filling questionnaire a flow chart may not be necessary. FUNAAB = 204 \| 75 (17.2); UI = 454 \| 73 (16.7); UNILORIN = 250 \| 26 (5.9); UNIABUJA = 392 \| 71 (16.2); UNIJOS = 190 \| 27 (6.2); UNIMAID = 500 \| 18 (4.1); UDUS = 500 \| 47 (10.8); ABU= 400 \| 14 (3.2); MOUAU = 250 \| 8 (1.8); UAM = 194 \| 60 (13.7); UNN = 390 \| 18 (4.1)  | | | |
| **Response to Reviewer 1** | | | |
| **Materials and Methods** | | | |
|  | Lines 131-133 | “The total sample size was divided equally among the veterinary schools (43 students per university). We purposively recruited a minimum of nine at each level (DVM 1-DVM 5) across the participating universities" – this is more like a Quota sampling; So the statement in 135 -136 “Purposive and convenience chain referral sampling technique was performed for student recruitment” needs to be rephrase | Thank you so much Sir. The authors quite agree with this suggestion. We have therefore rephrased to read “ |
|  | | | |
| **Reviewer 2** | | | |
| **Materials and Methods** | | | |
|  |  | Reviewer #2: Statistical methods used to calculate the sample size related to KAP study and descriptive statistics using percentages and means for KAP. It does not allow comparisons and regression analyses. This was suggested initially and was not fully addressed. Using only a limited number of participants from each school does not mean you can compare by year and school, for example. An online survey is suited to recruit a large number of participants as this is a method to overcome other validity issues. As well, you will never be sure that those who filled the survey are actually those intended to. | Yes, we agree that the formula we used is for survey and not a comparative study as we did not set out to compare in the first place, as observed in case control, cohort and random clinical trials.  However, by virtue of the outcome variables that were categorized, we decided to look at possible independent variables that could explain the observed categorization (into good or poor). |
